# Pattern of Clues: Evidence of Distinct DNA Methylation in Newborns of Smoking Women

**DOI:** 10.1289/ehp.120-a402a

**Published:** 2012-10-01

**Authors:** Wendee Nicole

**Affiliations:** Wendee Nicole, based in Houston, TX, has written for *Nature*, *Scientific American*, *National Wildlife*, and other magazines.

When pregnant women smoke, it affects their children’s health well beyond the womb, increasing their risk of obesity, cancer, respiratory illness, and lung disorders. To study the roots of these associations, a team of investigators assessed DNA methylation across more than 470,000 cytosine–guanine dinucleotide (CpG) sites in cord blood samples and found a highly significant association between maternal smoking and methylation [*EHP* 120(10):1425–1431; Joubert et al.]. They observed differential methylation not only in genes known to be involved in the detoxification of tobacco smoke but also in one gene not previously associated with smoking.

The team tested 1,062 cord blood samples from the Norwegian Mother and Child Cohort Study (MoBa). The average age of the MoBa mothers was 29.5 years, and 12.8% had blood plasma levels of cotinine (a nicotine metabolite) consistent with active smoking. The scientists analyzed 473,844 CpG sites across the genome using the 450K BeadChip assay platform and found differential methylation of 26 sites mapped to 10 different genes for smoking versus nonsmoking mothers. They then replicated these findings in a sample of 36 mothers from the North Carolina–based Newborn Epigenetics Study (NEST), half of whom reported smoking and half of whom did not.

Four of the 26 CpG sites were located on the aryl-hydrocarbon receptor repressor (*AHRR*) gene on chromosome 5, and several sites were just upstream of cytochrome P450 isoform *CYP1A1* on chromosome 15. Both genes are known to be involved in the aryl hydrocarbon receptor signaling pathway, which detoxifies polycyclic aromatic hydrocarbons, including those found in tobacco smoke. The MoBa cohort showed increasing methylation with cotinine levels in cord blood for one CpG site in the *AHHR* gene.

Growth factor independent 1 transcription repressor (*GFI1*) on chromosome 1 is a gene not previously associated with tobacco smoke, but in this study eight CpG sites on this gene were differentially methylated in children of smokers. *GFI1* plays a key role in several developmental processes, including the formation of blood cells, pulmonary neuroendocrine cells, and the inner ear. The gene also plays a role in cellular differentiation, proliferation, and oncogenesis.

**Figure:**
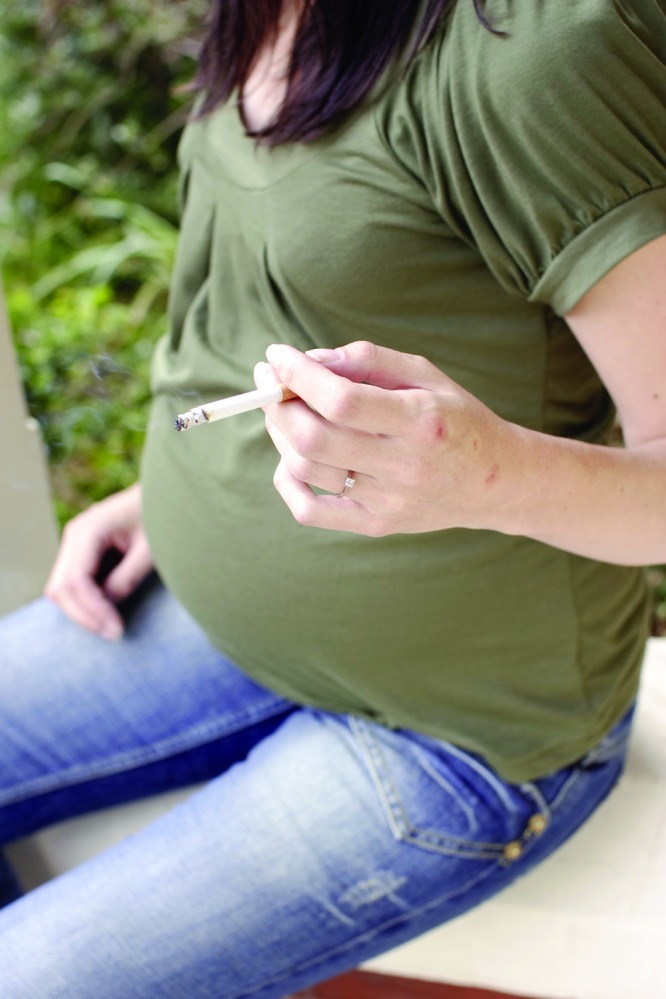
Researchers detected differential methylation of four genes in association with maternal smoking. © Bubbles Photolibrary/Alamy

The scientists found remarkable similarity in their results between the MoBa and NEST cohorts, both in terms of which genes were differentially methylated in relation to smoking and in the direction of the associations—in other words, whether smoking was related to increased or decreased methylation at a particular CpG site. Taken together, these two studies provide strong evidence for DNA methylation contributing to the effects of maternal smoking in children.

